# Randomized Trial to Assess the Safety and Tolerability of Daily Intake of an Allulose Amino Acid-Based Hydration Beverage in Men and Women

**DOI:** 10.3390/nu16111766

**Published:** 2024-06-05

**Authors:** Richard J. Bloomer, Jacquelyn Pence, Janine Hellenbrand, Allyson Davis, Samantha Davis, Michelle Stockton, Keith R. Martin

**Affiliations:** Center for Nutraceutical and Dietary Supplement Research, College of Health Sciences, University of Memphis, Memphis, TN 38152, USA; jpence1@memphis.edu (J.P.); j.hellenbrand@gmx.net (J.H.); laboswll@memphis.edu (A.D.); sdavis25@memphis.edu (S.D.); mstocktn@memphis.edu (M.S.); krmrtin4@memphis.edu (K.R.M.)

**Keywords:** hydration, electrolytes, amino acids, safety

## Abstract

Background: Maintaining adequate hydration is critical to optimal health, well-being, and performance. Those who are physically active in stressful environments, such as warm and/or humid scenarios, may be at particular risk for dehydration with ensuing loss of electrolytes, leading to sluggishness and impaired physical performance. Methods: We evaluated an electrolyte and amino acid product containing L-alanine and L-glutamine, as well as select vitamins [B3 (niacin), B5 (pantothenic acid), B6 (pyridoxine), B12 (cobalamin), and vitamin C (ascorbic acid)]. Subjects (*n* = 40; four groups, *n* = 10) were randomized to consume either a placebo packet or one, two, or three packets daily of the test product for 4 weeks with site visits at 0, 2, and 4 weeks. We tested safety and tolerability by analyzing hematological parameters (complete blood counts), metabolic parameters (hepatic, renal, acid–base balance), urinalysis end products, thyroid status [T3 (triiodothyronine), T4 (thyroxine), TSH (thyroid-stimulating hormone)], tolerability (via questionnaire), vital signs, and dietary intake. Results: Statistical analyses displayed ten significant main effects (*p* < 0.05) with white blood cells, lymphocytes, neutrophils, urinary pH, thyroxine, urination frequency, calcium, calories, fat, and cholesterol. Interactions for time and group (*p* < 0.05) were observed for MCV, eGFR, potassium, overall tolerability, bloating, and cramping—demonstrating mild GA disturbances. Little to no change of physiological relevance was noted for any outcome variable, regardless of dosing level. Conclusions: Our results indicate the product was well-tolerated at all dosing levels and no significant adverse changes occurred in any of the test parameters compared to the placebo group, indicating relative safety of ingestion over a 4-week treatment period, at the volumes used, and outside the context of physical stress.

## 1. Introduction

Maintaining adequate hydration is essential to optimal health [1], with these needs higher amongst those at greater risk of dehydration—including individuals who travel extensively, are exposed to extreme outdoor environments, and those who exercise regularly or who are highly active in general [2,3]. For example, individuals exercising in warm and/or humid environments can lose excessive amounts of fluids along with necessary electrolytes (e.g., sodium, potassium, chloride) through sweating, which may cause dehydration, sluggishness, and impaired physical performance [4]. Continued exacerbation of this state can lead to potentially life-threatening medical emergencies.

Consumption of electrolytes (sodium in particular) in beverages has been used for decades to aid the hydration of athletes and has led to the development of various sport drinks (e.g., Gatorade, Powerade) that have been specifically designed for this purpose. As well as containing small amounts of carbohydrates, the addition of non-essential amino acids has been demonstrated to further increase the transport of water and sodium from the jejunum (small intestine) into systemic circulation for redistribution within the body as needed [5,6]. Amino acids are certainly important for overall health [7], and prior research has investigated the impact of amino acids added to electrolyte-containing beverages to improve hydration status. While there is emerging interest in this approach, the hydration effects appear similar to those observed with a standard carbohydrate–electrolyte drink, with the possible exception of a quicker return to baseline in urine specific gravity for the amino-acid-containing beverage [8]. A more recent study [9] found that hydration with amino acid beverages increased the well-recognized beverage hydration index (BHI [10])—a measure of fluid balance following consumption of a beverage—which was age-dependent. Supplementation with amino acids has gained acceptance and increased usage by athletes due to other reported potential performance benefits including changes in anabolic hormone levels, alterations in energy consumption, and improvement of mental endurance [11,12,13]; moreover, supplemental amino acid consumption may reduce post-exercise muscle soreness and aid in recovery after numerous, diverse physical activities.

For this study, an electrolyte amino-acid test product was developed as a powder provided in individual “stick” packs to be dissolved in water and taken orally. Each stick pack contains the naturally occurring and near-zero calorie sweetener allulose, as well as the amino acids L-alanine and L-glutamine, the electrolytes sodium (510 mg, 22% DV) and potassium (380 mg, 8% DV), and five essential nutrients including vitamins B3 (niacin), B5 (pantothenic acid), B6 (pyridoxine), B12 (cobalamin), and vitamin C (ascorbic acid). The product is gluten-, soy-, and dairy-free and provides electrolytes and amino acids well below the daily value to minimize the potential for exceeding dietary levels that may cause adverse effects. Hence, it was hypothesized that multiple servings could be consumed daily without adverse events; however, this remained to be determined. Therefore, the aim of this work was to determine the safety of the product specific to blood counts and chemistries, vital signs, urinalysis, and quantification of T3 (triiodothyronine), T4 (thyroxine), and TSH (thyroid-stimulating hormone). The above variables were chosen, including the thyroid hormones, to best represent common clinical outcomes that are routinely used as a part of a comprehensive physical examination. A questionnaire was also used to assess product tolerability. It was hypothesized that daily ingestion of the product would not lead to any adverse effects specific to our outcome measures, regardless of dose, as the ingredients included within the product are quite benign and routinely consumed as a part of a standard diet (with the possible exception of allulose).

## 2. Materials and Methods

### 2.1. Subjects

Male and female subjects (*n* = 40; see Table 1 for details) were recruited from the University of Memphis campus and the local Memphis area via word of mouth, snowball sampling (chain referral sampling/recruiting), recruitment postings to various websites and social media channels, and flyers. As an initial study, no power analysis was performed and a modest sample size (10 subjects per group) was chosen to determine outcomes and to direct future larger-scale studies. This study recruited subjects between the ages of 18 and 50 years, as this is the anticipated primary age range of the consumer audience of hydration products. Subjects had a body mass index (BMI) between 20 and 29.9 kg/m^2^ (not obese), with higher values being excluded to reduce the variability in hydration status since individuals with obesity consume less water than those of normal weight [14,15]. Study inclusion was restricted to adult subjects who were healthy with no history of chronic health concerns such as a major affective disorder, cardiovascular disease, diabetes, and gastrointestinal disorders, as well as being smoke-free (e.g., non-tobacco or vape user). This study excluded female subjects who were pregnant or trying to become pregnant. Subjects refrained from alcohol and caffeine use at least 24 h prior to site visits and had no prior history of chemical abuse. Health histories and dietary records were completed by all subjects and reviewed by investigators. Subjects signed informed consent, as approved by the University Institutional Review Board for Human Subjects Research (Protocol # PRO-FY2023-5). Qualifying subjects were randomized into treatment groups using a balanced randomization for sex (see distribution in Table 1).

### 2.2. Test Product

The electrolyte amino-acid test product was a powder in individual stick pack form that contained a proprietary sugar-free and near-zero-calorie blend of 8–9 g of allulose, amino acids (l-alanine and l-glutamine), and electrolytes (510 mg of sodium, 380 mg potassium per stick pack) as well as vitamins B3, B5, B6, B12, and vitamin C. The product was gluten-, soy-, and dairy-free. The product (Liquid I.V. Hydration Multiplier Sugar-Free; Liquid I.V., El Segunda, CA, USA) was produced in accordance with Good Manufacturing Practices and packed in unlabeled packets.

### 2.3. Test Visit Procedures

**Figure 1:**
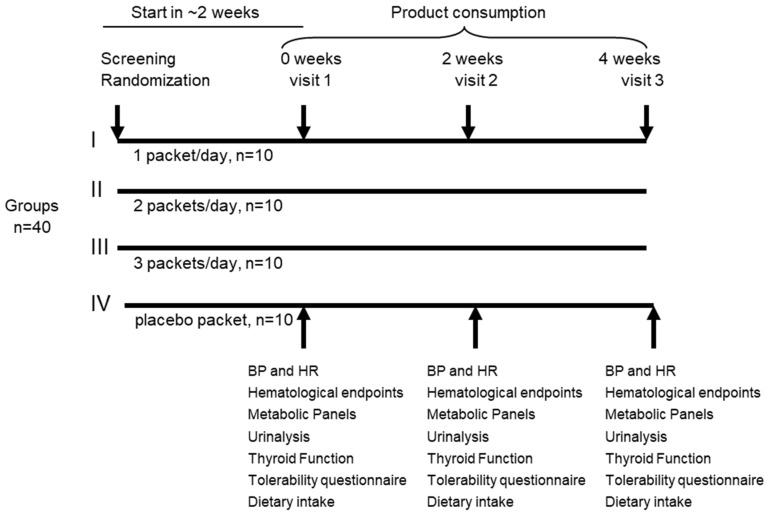
Experimental design of study.

At each visit, subjects reported to the study center in the early morning hours (e.g., 6:00–8:00 a.m.) in a 10-hour fasted state and self-collected a mid-stream urine sample for subsequent analysis. Subjects then rested quietly for 10 min before heart rate and blood pressure (systolic/diastolic) were taken using an automated point-of-care sphygmomanometer (OMRON HEM 907XL, OMRON Healthcare, Tokyo, Japan). No EKG assessment was performed, which may be considered a limitation of this study. Height and weight were measured using a calibrated platform scale with a stadiometer (TANITA, ELECTRONIC PHYSICIAN SCALE WB-3000, Tokyo, Japan). Subjects reviewed their medication/dietary supplement use with an investigator. A blood sample was collected and analyzed as described below. Subjects were randomized to one of four groups (See Figure 1) to consume either (1) one stick of product, (2) two sticks of product, (3) three sticks of product, or (4) one stick of flavor-matched and sugar-free Crystal Light (placebo) daily for four total weeks. Subjects were instructed to thoroughly mix the powder into 16 ounces of water and consume in the morning, afternoon, and/or evening, while in a rested state and not specific to an acute bout of exercise. During visit 2 (at the 2-week midpoint), the subject repeated the procedures and returned the remaining study product and their study journal. Subjects received a new study journal and study product for the second half of the study. At visit 3, subjects repeated the above procedures and completed a product tolerability questionnaire.

### 2.4. Physical Activity and Dietary Intake

Subjects followed their usual everyday activity patterns over the course of the study period and refrained from strenuous activity, alcohol, and caffeine for 24 h preceding each lab test day. To validate, all subjects were provided with a study journal to be completed during the 3 days prior to each visit and returned at the subsequent visit to monitor daily food and beverage intake, physical activity, compliance, and self-reported tolerability to the test product. Subjects were also asked to return any unused product at each visit as an indirect measure of compliance. Diet records were analyzed for macro- and micro-nutrients using Food Processor Pro software, version 11.11, with daily averages presented. The contents of the daily treatment were included in the analysis.

### 2.5. Urinalysis

At the study center, subjects provided a clean-catch urine sample at each visit to the study center. Mid-stream urine samples were collected by subjects in a private restroom within the study center at each visit and were passed into a standard urine collection container. Investigators used a standard over-the-counter urinalysis test strip kit (AccuMed hCG test) to determine pregnancy. A 10 mL urine sample was also collected for analyses (color, appearance, specific gravity, pH, protein, glucose, ketones, occult blood, leukocyte esterase, nitrite, bilirubin, and urobilinogen) at LabCorp. Inc, Burlington, NC, USA, using their standard laboratory procedures.

### 2.6. Blood Collection, Hematology, and Clinical Chemistries

Subjects provided a single sample of blood from the antecubital fossa (elbow crease) at each visit by standard venipuncture. Approximately 15 mL of blood (~1 tablespoon) was collected via a 21-gauge needle into sterile vacutainer tubes with either serum separator (SST)—for analysis of TSH, ALT, AST, albumin, alkaline phosphatase, total bilirubin, blood urea nitrogen (BUN), calcium, CO_2_, chloride, creatinine, eGFR (estimated glomerular filtration rate) calculation, glucose, potassium, total protein, sodium, total globulin, albumin–globulin (A–G) ratio, BUN–creatinine ratio, and T3 and T4 (at V1, V2, V3 only)— or EDTA preservative vacutainers—used for analysis of hematocrit, hemoglobin, mean corpuscular volume (MCV), mean corpuscular hemoglobin (MCH), mean corpuscular hemoglobin concentration (MCHC), red cell distribution width (RDW), percentage and absolute differential counts, platelet count, red blood cell count (RBC), and white blood cell count (WBC).

### 2.7. Statistical Analysis

Descriptive statistics were used to outline baseline characteristics (means, standard deviations, frequencies, and percentages) and analysis of variance tests were used to evaluate whether there were any significant differences between the groups on baseline variables, with Bonferroni post hoc analyses conducted on any significant findings. This study consisted of four groups (*n* = 10/group) at four different dose levels (0, 1, 2, and 3 sticks of study product) consumed daily for four weeks with lab visits at baseline, two, and four weeks. As such, statistical analyses involved two-way mixed ANOVAs to identify the presence of significant differences between groups. Appropriate Bonferroni post hoc analyses were conducted if significance was noted. Effect sizes were reported as partial eta-squared, where η^2^ = 0.01 indicates a small effect, η^2^ = 0.06 indicates a medium effect, and η^2^ = 0. 14 indicates a large effect. Outliers in the data were assessed by inspection of a boxplot and studentized residuals. Normality was assessed by a Normal Q–Q Plot and Shapiro–Wilk’s test (*p* > 0.05). Homogeneity of variances or covariances was assessed by Levene’s test of homogeneity of variances and Box’s M test, respectively (*p* > 0.05). Mauchly’s test of sphericity was used to ensure the assumption of sphericity was not violated for the two-way interaction. Statistically significant interactions between the treatment groups and time were determined for each test parameter or study outcome. Bonferroni correction was applied to adjust the significance level, and this is discussed below. All analyses were performed using the Statistical Package for the Social Sciences (SPSS for Windows, version 28; SPSS, Chicago, IL, USA). All statistical tests were two-tailed with the significance level represented by *p* < 0.05.

## 3. Results

A total of 40 men and women completed all aspects of this study, with baseline characteristics of anthropometric measures and vital signs (Table 1) and hematological and metabolic variables (Table 2) presented. It should be noted that when applying the Bonferroni correction, there were no variables within the dataset showing statistical significance. That said, because this is an exploratory study with a very large number of outcome measures, we choose to present the data and analysis without considering the Bonferroni correction.

With the above in mind, there were no baseline differences noted to be of statistical significance for the anthropometric measures and vital signs (*p* > 0.05). There were also no baseline differences in the majority of the hematological and metabolic measures between the four conditions, except for RDW (*p* = 0.03); however, post hoc tests revealed that none of the groups were statistically significantly different from one another (Table 3 and Table 4).

WBC visit 1 vs. visit 2 (*p* = 0.04); MCV Group 2 different across time (*p* = 0.03); eGFR, Group 3 different across time (*p* = 0.009); Calcium, difference between Groups 3 and 4 (*p* = 0.05).

*White Blood Cells (Differential; WBC).* The main effect of time showed statistically significant differences in mean WBC at the different time points, F(2, 70) = 3.46, *p* = 0.037, partial η^2^ = 0.090. Visit 1 was significantly different than visit 2 across all groups on WBC (MD = −0.416, SE = 0.163, *p* = 0.046).

*Mean Corpuscular Volume (MCV*). Simple main effects for time indicated that for Groups 1, 3, and 4, MCV was not statistically significantly different across the time points (*p* = 0.068, 0.235, and 0.159, respectively); however, treatment Group 2 was statistically significantly different across the time points (*p* = 0.032). There was a statistically significant interaction between the treatment groups and time for MCV, F(6, 70) = 2.75, *p* = 0.019, partial η^2^ = 0.191, although the simple main effects for the treatment group did not yield significant findings.

*Lymphocytes.* The main effect of time showed statistically significant differences in mean lymphocytes at the different time points, F(2, 70) = 3.84, *p* = 0.038, partial η^2^ = 0.099; however, pairwise comparisons did not show significant differences in time (visit 1 versus visit 2, *p* = 0.150; visit 1 versus visit 3, *p* = 1.00; visit 2 versus visit 3, *p* = 0.099).

*Neutrophils.* The main effect of time showed statistically significant differences in mean neutrophils at the different time points, F(2, 70) = 3.98, *p* = 0.031, partial η^2^ = 0.102; however, pairwise comparisons did not show significant differences in time (visit 1 versus visit 2, *p* = 0.166; visit 1 versus visit 3, *p* = 1.00; visit 2 versus visit 3, *p* = 0.063).

*Estimated Glomerular Filtration Rate (eGFR).* The simple main effects for the treatment group did not yield significant findings for visit 1, F(3, 36) = 0.511, *p* = 0.677, partial η^2^ = 0.041; for visit 2 F(3, 36) = 0.008, *p* = 0.999, partial η^2^ = 0.001; or for visit 3 F(3, 36) = 0.905, *p* = 0.448, partial η^2^ = 0.070. Simple main effects for time indicated that the eGFR in Groups 1, 2, and 4 were not statistically significantly different across the time points (*p* = 0.359, 0.260, and 0.159, respectively). Treatment Group 3 was statistically significantly different across the time points (*p* = 0.009). Pairwise comparisons showed a significant difference for Group 3 between visit 1 and visit 2 (*p* = 0.031). There was also a statistically significant interaction between the treatment groups and time for eGFR, F(6, 70) = 2.48, *p* = 0.031, partial η^2^ = 0.175.

*Thyroxine.* The main effect of treatment group did show statistically significant differences in mean thyroxine between intervention groups, F(3, 35) = 3.23, *p* = 0.034, partial η^2^ = 0.217. Pairwise comparisons showed a statistically significant difference between Groups 3 and 4 (*p* = 0.033). No other groups were statistically significantly different.

*Calcium.* The main effect of treatment group did show statistically significant differences in mean calcium between intervention groups, F(3, 35) = 3.13, *p* = 0.038, partial η^2^ = 0.212. Pairwise comparisons showed a statistically significant difference between Groups 1 and 3 (*p* = 0.050). No other groups were statistically significantly different.

*Potassium.* There was a statistically significant interaction between the treatment groups and time on potassium, F(6, 70) = 2.55, *p* = 0.027, partial η^2^ = 0.107. Simple main effects for the treatment group indicated that there was a statistically significant difference in potassium at visit 1, F(3, 36) = 3.35, *p* = 0.030, partial η^2^ = 0.218. There was not a statistically significant difference in potassium between groups at visit 2, F(3, 36) = 0.346, *p* = 0.793, partial η^2^ = 0.028, nor at visit 3, F(3, 36) = 1.79, *p* = 0.167, partial η^2^ = 0.129. Simple main effects for time indicated that for Groups 1, 2, and 4, potassium was not statistically significantly different across the time points (*p* = 0.167, 0.127, and 0.864, respectively). Group 3 was statistically significantly different across the time points, F(2, 18) = 8.77, *p* = 0.002, partial η^2^ = 0.494, with visit 1 being lower than visit 2 (*p* = 0.023) and visit 1 being lower than visit 3 (*p* = 0.008).

*Urinary pH.* The main effect of time showed statistically significant differences in mean pH at the different time points, F(2, 70) = 6.99, *p* = 0.002, partial η^2^ = 0.163. Pair-wise comparisons showed statistically significant differences in time between visit 1 and visit 2 (*p* = 0.001). The other visit comparisons were not statistically significant (visit 1 and visit 3, *p* = 0.056; visit 2 and visit 3, *p* = 0.678). The main effect of treatment group did not show statistically significant differences in mean pH between intervention groups, F(3, 35) = 0.429, *p* = 0.734, partial η^2^ = 0.034.

*Total Tolerability Score.* There was a statistically significant interaction between the treatment groups and time on total tolerability, F(1.54, 49.28) = 2.49, *p* = 0.044, partial η^2^ = 0.193; although interactions were significant, the simple main effects for the treatment group and for time did not yield significant findings.

*Bloating.* There was a statistically significant interaction between the treatment groups and time on bloating, F(4.895, 53.842) = 2.56, *p* = 0.039, partial η^2^ = 0.189; although interactions were significant, the simple main effects for the treatment group and for time yielded no significant findings.

*Abdominal Cramping.* There was a statistically significant interaction between the treatment groups and time on abdominal cramping, F(6, 66) = 3.54, *p* = 0.010 partial η^2^ = 0.243. Simple main effects for the treatment group indicated that there was a statistically significant difference in abdominal cramping at visit 2, F(3, 36) = 3.74, *p* = 0.019, partial η^2^ = 0.238.

*Frequent Urination*. The main effect of time was statistically different in mean frequency of urination at the different time points (*p* = 0.007). Pairwise comparisons showed statistically significant differences in time between visit 1 and visit 2 (*p* = 0.004).

Data for the above variables are presented in Table 5.

Dietary data are presented in Table 6. 

*Calories.* The main effect of time was statistically significantly different in mean calories at the different time points, F(2, 72) = 4.13, *p* = 0.020, partial η^2^ = 0.103.

*Fat.* The main effect of time was statistically significant in mean fat at the different time points, F(2, 72) = 4.28, *p* = 0.021, partial η^2^ = 0.106.

*Cholesterol.* There was a statistically significant interaction between the treatment groups and time on cholesterol, F(6, 72) = 3.35, *p* = 0.006, partial η^2^ = 0.218. Simple main effects for the treatment group indicated that there was a statistically significant difference in cholesterol at visit 1, F(3, 36) = 3.35, *p* = 0.030, partial η^2^ = 0.218. There was not a statistically significant difference in cholesterol between groups at visit 2, F(3, 36) = 0.346, *p* = 0.793, partial η^2^ = 0.028, nor at visit 3, F(3, 36) = 1.79, *p* = 0.167, partial η^2^ = 0.129. Simple main effects for time indicated that for Groups 1 and 2, cholesterol was not statistically significantly different across the time points (*p* = 0.253 and 0.110, respectively). Group 3 was statistically significantly different across the time points, F(2, 18) = 3.63, *p* = 0.047, partial η^2^ = 0.288, with visit 1 being higher than visit 2 (*p* = 0.011). Group 4 was statistically significantly different across the time points, F(2, 18) = 4.36, *p* = 0.029, partial η^2^ = 0.326; however, none of the pairwise comparisons were significantly different.

## 4. Discussion

We evaluated the safety and tolerability of an allulose-containing amino acid hydration powder in healthy men and women. With dietary intake remaining relatively constant across the study period, the beverage was well-tolerated and did not result in any adverse outcomes. While select bloodborne variables were slightly different across time or between groups, these did not appear to be influenced by treatment (i.e., lack of interactions). Rather, the findings of statistical significance were likely due to a small change in mean concentrations, coupled with the relatively low variability in response due to the tight physiological regulation of these variables.

Maintaining adequate hydration is critical to optimal health and can prove particularly helpful to those who travel extensively, are exposed to extreme outdoor environments, and who exercise regularly. For example, multiple studies have focused specifically on the effects of dehydration on soldier performance, particularly in desert environments [16]. Occupational exposure to high temperatures, whether indoors or outdoors, can lead to dehydration and puts workers at a greater risk of chronic kidney disease [17]. In this study, we tested the safety and tolerability of an electrolyte- and amino acid-supplemented product, developed as a powder and provided in individual “stick” packs to be dissolved in water and consumed orally.

Although electrolytes are commonly employed to promote hydration, amino acids can further enhance the absorption of sodium and water via multiple transporters in the small intestine [18]. To this end, studies have demonstrated that incorporating amino acids into electrolyte beverages can support hydration in a similar manner as a carbohydrate–electrolyte beverage [8,9]. This may be of interest to those who choose to limit carbohydrate intake, such as individuals following a ketogenic diet. Further, amino acids have been suggested as a potential alternative to carbohydrates/glucose in beverages [18,19], with additional benefits for other aspects of health [8]. While we noted some statistically significant effects for select variables, from a physiological perspective, none were altered at a level of concern, even when subjects ingested the highest dosage of three packets per day for four weeks. These findings indicate the product was well-tolerated at all test levels and no significant adverse changes occurred in any of the test parameters. To our knowledge, this is the first evaluation of this test product pertaining to safety.

There were statistical differences in means for certain hematological parameters. For example, white blood cell numbers were significantly increased for all groups at visit 2 compared with visit 1 (baseline or zero) but there were no significant differences between treatment groups nor were there any two-way interactions. At the different time points, there were statistically significant differences in mean lymphocytes, which is likely due to interactions of time and treatment and not either one alone. For red blood cells, the MCV in Groups 1, 3, and 4 were not statistically significantly different across the time points; however, Group 2 was statistically significantly different across the time points (p = 0.032). The values for this group started initially much lower than the other treatment groups and remained so throughout the duration of the study. In fact, pair-wise comparisons of the three groups were not significant (*p* > 0.05) for any of the treatment groups.

Significant differences in mean thyroxine levels were observed between intervention groups. Pair-wise comparisons showed a statistically significant difference between Groups 3 and 4 (*p* = 0.033) but not for other groups. It appears that a transient spike in thyroxine occurred at visit 2 for Group 3. Thyroxine is the primary hormone secreted into the bloodstream by the thyroid gland. Although less active than other chemical forms, the majority is converted to triiodothyronine (T3), a more active form, by the liver, kidneys, skeletal and heart muscles, central nervous system, skin, etc. There are numerous potential explanations for a transient spike in thyroxine levels including acute illness, dietary changes, and/or dietary supplement use such as biotin (B vitamin) supplementation, which can lower TSH levels. An alternative explanation for a transient spike is that the data may be spurious and the effect, although statistically significant, may not be biologically relevant or intrinsically valid.

A cross-sectional study of 132,346 male and female participants [20] with normal thyroid function within a single institution determined that both the FT3:FT4 ratio and TSH were positively associated with markers for insulin resistance and parameters of metabolic syndrome (MetS). Specifically, FT3:FT4 positively correlated with HOMA-IR, waist circumference, triglyceride levels, fasting blood glucose, and systolic blood pressure. Interestingly, the FT3 to FT4 ratio had a stronger association with MetS risk than did TSH alone [20]. In our study, individuals with BMIs of 25.0–29.9 kg/m^2^—categorized as overweight—were enrolled and could have introduced variance in measured endpoints. Given this propensity for fluctuation, TSH is the most used initial screening test for thyroid dysfunction since changes in TSH occur much earlier than changes in T3/T4. In our study, TSH levels ranged from 1.57 to 2.34 mIU/L. Typically, if values are outside the range of 0.40–4.50 mIU/L for TSH, measuring T3 and T4 would follow in a clinical setting. Thus, in our study, all values for TSH at all time points and groups were within normal clinical range. Interestingly, the reported normal T4:T3 ratio is around 13:1, which corresponds to Group 3 where a statistically significant difference in thyroxine was noted. Other ratios were higher than normal [21].

There were some notable differences in mineral/electrolyte levels. There were statistically significant differences in mean calcium between intervention groups (*p* = 0.038); however, the differences are physiologically irrelevant (Table 3). Pair-wise comparisons showed a statistically significant difference between Groups 1 and 3 (*p* = 0.050) but not in other groups. There was a statistically significant difference in potassium levels at visit 1 (*p* = 0.0030) but no statistically significant differences between groups at visit 2 nor at visit 3. There was a statistically significant interaction between the treatment groups and time on potassium, which is not surprising considering the ingestion of the treatment.

pH measurements are an indicator of the acid–base balance in the bloodstream and are fairly well-controlled at a range of 7.35–7.45. Although pH was not measured in the blood in this study, we did measure urinary pH and values were consistent between groups, with a slight change noted across time—a statistically significant difference between visit 1 and visit 2 (*p* = 0.001). Future work may choose to measure pH in the blood—in particular, if using the treatments in the context of physical exercise. It has been reported that there are three independent variables that regulate pH in blood plasma including carbon dioxide, relative electrolyte concentrations, and total weak acid concentrations; furthermore, all pH changes, whether in health or disease, are via these three variables [22]. It is possible that the changes in electrolytes in this study (sodium and potassium), which were statistically significant, may have contributed to changes in pH.

eGFR is an indirect indicator of renal function and urinary output based on serum creatinine. Groups 1, 2, and 4 were not statistically significantly different across the time points (*p* = 0.359, 0.260, and 0.159, respectively); however, treatment Group 3 was statistically significantly different across the time points (*p* = 0.009) and specifically between visit 1 and visit 2 (*p* = 0.031). There was also a statistically significant interaction between the treatment groups and time on eGFR.

There were significant results noted in total tolerability, bloating, abdominal cramping, and urination frequency. Although there were no statistically significant differences in total tolerability between groups at any of the visits, there was a statistically significant interaction between the treatment groups and time on total tolerability. The same observation was noted for bloating. However, the simple main effects for the treatment group and for time yielded no significant findings. There were no statistically significant differences in bloating between groups nor for time among any of the treatment groups. There was, however, a statistically significant interaction between the treatment groups and time on abdominal cramping (*p* = 0.010), which appeared to be influenced mainly by the data of one individual who reported high values across times. Simple main effects for the treatment group indicated that there was a statistically significant difference in abdominal cramping at visit 2 (*p* = 0.019) but not for groups at visit 1 nor at visit 3. Abdominal cramping was not different at any of the time points (*p* = 0.306). Collectively, it appeared that the total tolerability and various other outcomes depended on both time and dose, viz., group, as an interaction for significant differences to become evident. Neither time nor dose alone caused significant differences amongst outcomes. It is possible that the addition of allulose to the product may have been at least partly responsible for these findings, as allulose has been reported to promote GI disturbances. That said, it should be noted that very high dosages of allulose are typically needed (i.e., 30 g) to yield such problems [23]. If the product is used in the context of physical exercise and the dosage is increased considerably beyond what was used in the present study, the potential for GI distress may be increased; however, future work is needed to confirm this hypothesis.

Mean frequent urination was different amongst the time points (*p* = 0.007), with pair-wise comparisons showing time-dependent significant differences between visit 1 and visit 2 (*p* = 0.004). Other visit comparisons were not statistically significant (visit 1 and visit 3, *p* = 0.560; visit 2 and visit 3, *p* = 0.083). Mean frequent urination between intervention groups (*p* = 0.313) did not show statistically significant differences as did the treatment groups and time (*p* = 0.386). Interestingly, most of the noted change across visits was for the two packets per day dosage, suggesting that changes perhaps were not so much influenced by the product but by certain subjects who happened to be assigned to that group. As with many food-based products, individual responses can vary considerably, and this may have been the case in the present study—with a few individuals driving the majority of the effects in terms of tolerability findings.

Pertaining to dietary intake, there were differences noted in select variables, as indicated in Table 6. It is possible that the addition of amino acids and other nutrients to the drink may have influenced the dietary intake of participants. That said, although values were noted as being of statistical significance, when looking carefully at the data across time and between treatment groups, there is little physiological relevance. More research is needed to determine what, if any, influence this electrolyte–amino acid formula may have on dietary intake in men and women.

## 5. Conclusions

We evaluated the impact of an electrolyte–amino acid drink, at three different dosages, on a variety of safety parameters in 40 men and women. Our results indicate the product was well-tolerated at all dosing levels, with no significant adverse events noted, albeit minor increases in certain GI disturbances. When considering the 4-week treatment period as used in the present study, the product appears safe for human consumption— outside the context of physical stress—even at the relatively high dosage of three stick packs per day.

## Figures and Tables

**Table 1:** Baseline subject characteristics by condition [mean (SD) or *n* (%)] *.

Characteristics	Group 1 1 Stick (*n* = 10)	Group 2 2 Sticks (*n* = 10)	Group 3 3 Sticks (*n* = 10)	Group 4 Placebo (*n* = 10)
Age (yrs)	24.3 (8.7)	25.4 (4.7)	27.2 (8.1)	32.8 (9.5)
Sex, *n* (%)				
Male	4 (40)	4 (40)	4 (40)	4 (40)
Female	6 (60)	6 (60)	6 (60)	6 (60)
Height (cm)	168.5 (8.4)	168.6 (8.7)	171.8 (9.7)	167.2 (8.1)
Weight (kg)	68.2 (12.1)	64.3 (8.4)	72.4 (9.3)	68.5 (14.4)
BMI (kg/m^2^)	23.9 (2.5)	22.6 (1.4)	24.5 (2.2)	24.3 (3.1)
Waist (cm)	78.6 (7.2)	75.2 (6.6)	79.0 (7.3)	80.0 (12.6)
Hip (cm)	99.1 (4.7)	97.3 (5.1)	100.9 (5.4)	99.4 (5.4)
Systolic Blood Pressure (mm Hg)	114.3 (8.1)	108.8 (14.8)	117.4 (8.5)	111.6 (11.9)
Diastolic Blood Pressure (mm Hg)	73.2 (4.5)	70.2 (10.1)	74.0 (7.4)	74.9 (9.6)
Heart Rate (bpm)	76.1 (8.8)	72.7 (16.6)	64.6 (9.5)	72.2 (12.2)
Temperature (°C)	36.7 (0.2)	36.7 (0.2)	36.7 (0.2)	36.7 (0.2)

* No variable had statistical significance between groups (*p* > 0.05).

**Table 2:** Baseline subject characteristics by condition [mean (SD) or *n* (%)].

Characteristics	Group 1 1 Stick (*n* = 10)	Group 2 2 Sticks (*n* = 10)	Group 3 3 Sticks (*n* = 10)	Group 4 Placebo (*n* = 10)
WBC (×10^3^/μL)	6.0 (1.6)	5.5 (0.8)	5.6 (1.4)	5.3 (1.9)
RBC (×10^6^/μL)	4.8 (0.6)	4.7 (0.4)	4.8 (0.4)	4.6 (0.4)
Glucose (mg/dL)	89.6 (9.4)	86.2 (3.8)	87.5 (5.4)	89.3 (9.3)
Hemoglobin (g/dL)	13.8 (1.7)	13.9 (0.5)	13.6 (1.5)	13.8 (1.0)
Hematocrit (%)	42.1 (4.0)	41.9 (2.1)	41.7 (3.7)	41.5 (2.6)
MCV (fL)	89.0 (6.3)	89.7 (4.2)	86.9 (3.6)	90.8 (5.3)
MCH (pg)	29.1 (2.5)	29.9 (2.0)	28.3 (1.8)	30.1 (2.3)
MCHC (g/dL)	32.8 (1.0)	33.3 (0.9)	32.6 (1.0)	33.2 (1.0)
RDW (%) **	13.2 (1.1)	12.3 (0.7)	13.1 (0.8)	12.3 (0.7)
Platelets (×10^3^/μL)	270.2 (80.8)	252.9 (68.4)	262.7 (49.7)	259.1 (69.2)
Neutrophils (%)	51.2 (12.0)	55.5 (7.7)	52.2 (7.7)	50.7 (6.8)
Lymphocytes (%)	39.1 (11.2)	33.4 (7.3)	36.8 (7.2)	37.5 (5.2)
Monocytes (%)	6.8 (1.5)	7.7 (0.9)	7.8 (1.5)	8.2 (1.9)
Eos (%)	1.8 (0.9)	2.8 (1.6)	2.0 (0.7)	2.7 (2.2)
Basos (%)	1.1 (0.3)	0.6 (0.5)	1.0 (0.5)	0.9 (0.6)
Neutrophils (Absolute) (×10^3^/μL)	3.2 (1.4)	3.0 (0.6)	2.9 (0.9)	2.7 (1.1)
Lymphs (Absolute) (×10^3^/μL)	2.2 (0.7)	1.8 (0.5)	2.1 (0.7)	2.0 (0.7)
Monocytes (Absolute) (×10^3^/μL)	0.4 (0.1)	0.4 (0.1)	0.5 (0.1)	0.4 (0.1)
Eos (Absolute) (×10^3^/μL)	0.1 (0.1)	0.2 (0.1)	0.1 (0.03)	0.1 (0.1)
Baso (Absolute) (×10^3^/μL)	0.04 (0.1)	0.01 (0.03)	0.05 (0.05)	0.04 (0.05)
Immature Granulocytes (%)	0.0 (0.0)	0.0 (0.0)	0.0 (0.0)	0.0 (0.0)
Immature Granulocytes (Absolute) (×10^3^/μL)	0.0 (0.0)	0.0 (0.0)	0.0 (0.0)	0.0 (0.0)
BUN (mg/dL)	11.2 (2.5)	13.3 (4.0)	13.2 (4.9)	11.0 (2.9)
Creatine (mg/dL)	0.8 (0.2)	0.8 (0.2)	0.9 (0.2)	0.8 (0.2)
eGFR (mL/min/1.73)	115.2 (24.9)	108.3 (19.6)	105.7 (19.9)	109.8 (14.2)
BUN–Creatinine ratio	14.2 (3.0)	16.0 (3.9)	14.5 (2.8)	14.0 (3.7)
Sodium (mmol/L)	140.2 (1.5)	139.0 (2.7)	139.7 (1.4)	141.7 (2.8)
Potassium (mmol/L)	4.6 (0.4)	4.7 (0.5)	4.9 (0.4)	4.5 (0.5)
Chloride (mmol/L)	102.5 (1.7)	102.6 (2.3)	102.3 (1.2)	103.4 (2.7)
Carbon Dioxide (mmol/L)	23.1 (1.9)	23.7 (1.6)	24.3 (1.2)	23.8 (2.7)
Calcium (mg/dL)	9.6 (0.3)	9.5 (0.4)	9.7 (0.3)	9.6 (0.3)
Protein (g/dL)	7.2 (0.4)	6.8 (0.3)	7.2 (0.3)	7.1 (0.4)
Albumin (g/dL)	4.5 (0.3)	4.6 (0.3)	4.7 (0.4)	4.6 (0.2)
Globulin (g/dL)	2.6 (0.3)	2.2 (0.2)	2.5 (0.3)	2.5 (0.4)
A–G ratio	1.8 (0.3)	2.1 (0.3)	2.0 (0.3)	1.9 (0.3)
Alkaline Phosphatase (IU/L)	58.6 (16.8)	64.2 (17.1)	60.1 (18.2)	61.2 (16.0)
AST (SGOT) (IU/L)	17.4 (3.9)	22.3 (7.8)	18.4 (3.8)	23.0 (9.4)
ALT (SGPT) (IU/L)	12.9 (7.1)	18.0 (8.4)	13.8 (3.0	18.7 (9.3)
TSH (μIU/mL)	1.7 (0.5)	2.0 (0.7)	1.8 (0.7)	1.8 (0.8)

** *p* < 0.05; post hoc analyses showed no differences between the groups.

**Table 3:** Blood pressure, heart rate, temperature, and bloodborne data per visit across groups.

Outcome	Group 1		Group 2		Group 3		Group 4	
Measurement	1 Stick		2 Sticks		3 Sticks		Placebo	
	Mean	SD	Mean	SD	Mean	SD	Mean	SD
SBP (mmHg)								
Visit 1	116	7	106	8	111	12	111	10
Visit 2	112	10	109	6	112	10	104	10
Visit 3	111	11	111	10	116	11	108	8
DBP (mmHg)								
Visit 1	76	3	64	7	69	9	71	7
Visit 2	70	3	67	7	70	7	69	10
Visit 3	71	5	65	9	72	8	69	9
HR (bpm)								
Visit 1	78	10	67	8	69	13	73	14
Visit 2	78	12	65	8	67	11	71	19
Visit 3	74	8	66	6	66	13	72	13
Temperature (C)								
Visit 1	36.6	0.2	36.5	0.3	36.5	0.4	36.5	0.2
Visit 2	36.3	0.6	36.6	0.2	36.6	0.2	36.6	0.3
Visit 3	36.6	0.3	36.7	0.2	36.6	0.2	36.5	0.7
WBC (×10^3^/μL)								
Visit 1	5.27	1.03	5.19 *	0.99	5.08 *	1.29	5.18 *	1.85
Visit 2	5.78	1.35	5.35	1.02	5.93	2.04	5.49	1.72
Visit 3	5.05	1.10	5.29	1.23	5.82	2.15	5.20	1.66
RBC (×10^6^/μL)								
Visit 1	4.77	0.76	4.69	0.41	4.68	0.31	4.56	0.46
Visit 2	4.66	0.60	4.56	0.46	4.68	0.33	4.52	0.40
Visit 3	4.73	0.66	4.70	0.35	4.62	0.33	4.51	0.48
Hemoglobin (g/dL)								
Visit 1	13.81	1.68	13.82	0.68	13.31	1.24	13.59	0.92
Visit 2	13.50	1.57	13.52	0.78	13.32	1.18	13.58	0.99
Visit 3	13.65	1.58	13.94	0.87	13.19	1.23	13.45	1.10
Hematocrit (%)								
Visit 1	42.11	4.98	42.21	2.28	40.74	3.38	41.30	2.69
Visit 2	41.70	4.66	41.13	2.65	41.05	2.63	41.16	2.80
Visit 3	41.92	4.38	42.86	2.58	40.32	2.72	41.10	3.39
MCV (fL)								
Visit 1	89.00	6.78	90.40 *	5.38	87.10	3.45	91.20	5.81
Visit 2	90.10	6.95	90.60 *	4.60	87.90	4.51	91.33	5.59
Visit 3	89.10	6.51	91.60 *	4.97	87.40	4.48	91.60	5.48
MCH (pg)								
Visit 1	29.21	2.86	29.62	1.91	28.45	1.52	29.95	2.28
Visit 2	29.10	2.34	29.80	2.18	28.48	2.04	30.13	1.97
Visit 3	29.03	2.46	29.74	1.73	28.56	2.16	29.97	2.02
MCHC (g/dL)								
Visit 1	32.83	1.45	32.75	0.57	32.65	0.98	32.92	0.56
Visit 2	32.37	0.96	32.90	0.91	32.41	0.99	32.99	1.15
Visit 3	32.53	0.82	32.53	0.44	32.66	1.07	32.74	0.99
RDW (%)								
Visit 1	13.19	1.13	12.25	0.57	13.02	0.65	12.48	0.72
Visit 2	13.07	1.28	12.27	0.52	13.12	0.67	12.41	0.83
Visit 3	12.97	1.16	12.22	0.55	12.99	0.64	12.50	0.67
Platelets (×10^3^/μL)								
Visit 1	262.50	72.78	242.40	59.24	266.10	55.70	256.00	54.64
Visit 2	284.40	74.97	251.00	69.56	264.90	61.11	249.89	57.86
Visit 3	270.10	73.12	247.20	65.67	263.40	61.75	259.90	66.80
Neutrophils (%)								
Visit 1	49.9	10.0	52.7	6.2	48.8	8.8	49.1	6.1
Visit 2	51.4	11.8	52.4	10.4	54.8	6.6	53.1	8.3
Visit 3	48.9	9.5	51.8	6.9	51.3	10.0	48.1	7.0
Lymphocytes (%)								
Visit 1	38.8	9.5	35.9	6.5	39.9	8.2	38.6	5.5
Visit 2	38.8	10.8	36.3	7.6	34.2	6.0	33.8	9.0
Visit 3	40.0	9.8	36.2	5.0	37.4	9.0	39.2	6.1
Monocytes (%)								
Visit 1	7.7	1.6	8.1	1.6	8.0	1.8	8.6	1.4
Visit 2	6.9	1.9	7.7	1.9	7.9	1.7	8.9	1.5
Visit 3	7.3	1.9	7.9	2.0	8.1	1.7	7.9	1.1
Eos (%)								
Visit 1	2.3	1.2	2.6	1.4	2.2	0.9	2.8	1.8
Visit 2	2.0	1.4	2.9	2.2	2.0	0.7	3.2	1.7
Visit 3	2.6	1.2	3.3	2.2	2.1	1.0	3.0	1.8
Basos (%)								
Visit 1	1.1	0.3	0.7	0.5	1.0	0.0	0.9	0.6
Visit 2	0.9	0.3	0.7	0.5	0.9	0.3	0.9	0.8
Visit 3	1.1	0.3	0.7	0.5	1.0	0.0	0.8	0.6
Neutrophils Absolute (×10^3^/μL)								
Visit 1	2.74	0.92	2.69	0.42	2.52	0.84	2.58	0.90
Visit 2	3.07	1.19	2.88	0.92	3.29	1.19	3.04	1.44
Visit 3	2.52	0.78	2.79	0.86	3.10	1.61	2.59	0.92
Lymphocytes Absolute (×10^3^/μL)								
Visit 1	1.99	0.36	1.90	0.65	2.02	0.62	2.02	0.91
Visit 2	2.16	0.53	1.89	0.41	2.04	0.88	1.77	0.47
Visit 3	1.98	0.54	1.90	0.46	2.07	0.70	2.01	0.71
Monocytes Absolute (×10^3^/μL)								
Visit 1	0.41	0.09	0.43	0.07	0.41	0.10	0.41	0.14
Visit 2	0.39	0.14	0.40	0.05	0.46	0.20	0.49	0.12
Visit 3	0.38	0.15	0.41	0.09	0.46	0.15	0.42	0.12
Eos Absolute (×10^3^/μL)								
Visit 1	0.13	0.08	0.16	0.10	0.12	0.06	0.13	0.09
Visit 2	0.13	0.08	0.16	0.10	0.12	0.04	0.19	0.11
Visit 3	0.14	0.08	0.16	0.07	0.14	0.05	0.17	0.12
Baso Absolute (×10^3^/μL)								
Visit 1	0.05	0.05	0.03	0.05	0.06	0.05	0.03	0.05
Visit 2	0.05	0.05	0.03	0.05	0.08	0.04	0.03	0.05
Visit 3	0.05	0.05	0.02	0.04	0.06	0.05	0.03	0.05
Immature Granulocytes (%)								
Visit 1	0.2	0.42	0	0	0.1	0.32	0	0
Visit 2	0	0	0	0	0.2	0.42	0.11	0.33
Visit 3	0.1	0.32	0.1	0.32	0.1	0.32	0.1	0.32
Immature Granulocytes Absolute (×10^3^/μL)								
Visit 1	0	0	0	0	0	0	0	0
Visit 2	0	0	0	0	0.01	0.03	0	0
Visit 3	0	0	0	0	0	0	0	0
Glucose (mg/dL)								
Visit 1	89.90	5.22	86.30	6.00	84.90	5.97	90.20	8.74
Visit 2	90.80	13.42	88.00	5.72	85.50	7.41	88.67	9.42
Visit 3	89.20	8.89	85.00	6.46	83.50	4.25	86.40	8.33
BUN (mg/dL)								
Visit 1	12.70	4.11	14.10	2.89	13.40	3.84	12.40	3.81
Visit 2	11.50	4.09	14.20	3.08	14.30	2.63	11.56	3.54
Visit 3	13.00	3.71	14.40	4.01	15.00	4.76	12.60	3.10
Creatinine (mg/dL)								
Visit 1	0.83	0.25	0.89	0.19	0.91	0.26	0.82	0.20
Visit 2	0.85	0.25	0.82	0.17	0.85	0.21	0.74	0.15
Visit 3	0.83	0.24	0.85	0.12	0.90	0.23	0.78	0.14
eGFR (mL/min/1.73)								
Visit 1	113.5	21.309	105.3	17.134	103.70 *	19.574	109.2	19.194
Visit 2	110.6	21.573	111.6	15.204	110.80 *	18.183	110.44	17.436
Visit 3	113.5	21.046	108.3	13.325	103.60 *	19.34	114.7	12.544
BUN–Creatinine ratio								
Visit 1	16.10	6.74	16.00	2.26	15.10	3.54	15.50	4.43
Visit 2	14.40	6.17	17.50	3.24	17.30	2.67	15.67	3.94
Visit 3	16.70	6.55	16.80	4.08	16.50	2.37	16.40	3.13
Sodium (mmol/L)								
Visit 1	140.50	2.42	142.00	2.31	140.30	1.89	141.00	3.09
Visit 2	140.90	2.42	140.90	1.79	140.30	2.31	139.11	2.52
Visit 3	140.20	1.75	140.40	2.84	139.90	1.10	140.70	1.95
Potassium (mmol/L)								
Visit 1	4.58	0.24	4.59	0.49	4.71	0.28	4.73	0.57
Visit 2	4.62	0.34	4.50	0.29	4.57	0.33	4.46	0.27
Visit 3	4.82	0.40	4.57	0.32	4.74	0.36	4.62	0.49
Chloride (mmol/L)								
Visit 1	103.10	2.60	104.40	2.84	103.30	1.57	103.20	2.90
Visit 2	103.70	1.77	103.30	1.83	102.40	1.78	102.56	2.24
Visit 3	103.70	1.77	102.80	2.15	103.00	2.36	103.10	2.69
Carbon Dioxide (mmol/L)								
Visit 1	23.70	2.98	24.20	1.03	23.80	2.35	23.80	2.66
Visit 2	23.50	2.59	22.60	1.90	24.10	1.79	23.22	2.59
Visit 3	23.60	1.84	23.80	1.55	24.50	1.90	23.70	1.89
Calcium (mmol/L)								
Visit 1	9.49	0.36	9.55	0.24	9.60 *	0.18	9.46	0.42
Visit 2	9.49	0.16	9.43	0.35	9.51 *	0.28	9.27	0.24
Visit 3	9.48	0.19	9.47	0.33	9.46 *	0.21	9.35	0.29
Protein (g/dL)								
Visit 1	7.09	0.44	6.80	0.34	6.94	0.16	6.98	0.32
Visit 2	6.92	0.32	6.71	0.44	7.00	0.39	6.84	0.23
Visit 3	7.03	0.38	6.70	0.55	6.87	0.26	6.96	0.40
Albumin (g/dL)								
Visit 1	4.61	0.35	4.50	0.29	4.55	0.36	4.63	0.20
Visit 2	4.45	0.21	4.50	0.23	4.62	0.33	4.52	0.15
Visit 3	4.48	0.30	4.52	0.43	4.46	0.29	4.51	0.29
Globulin (g/dL)								
Visit 1	2.48	0.41	2.30	0.33	2.39	0.38	2.35	0.30
Visit 2	2.47	0.31	2.21	0.39	2.38	0.37	2.32	0.28
Visit 3	2.55	0.34	2.18	0.43	2.41	0.20	2.45	0.34
A–G ratio								
Visit 1	1.91	0.38	2.01	0.36	1.95	0.45	2.00	0.27
Visit 2	1.83	0.26	2.09	0.36	2.00	0.37	1.99	0.28
Visit 3	1.79	0.29	2.16	0.51	1.87	0.24	1.88	0.29
Alkaline Phosphatase (IU/L)								
Visit 1	59.00	13.20	64.70	16.01	60.00	17.24	62.00	14.40
Visit 2	54.80	15.22	59.70	18.01	55.50	15.97	62.56	15.35
Visit 3	56.50	16.70	58.10	14.96	53.60	15.99	63.00	16.79
AST SGOT (IU/L)								
Visit 1	19.60	6.08	19.40	3.66	17.00	4.55	21.00	7.42
Visit 2	18.90	7.78	20.30	4.90	19.20	3.52	21.44	6.04
Visit 3	17.60	4.09	21.00	7.27	23.00	16.46	20.80	7.67
ALT SGPT (IU/L)								
Visit 1	17.50	8.75	13.90	3.67	13.00	3.68	15.00	4.60
Visit 2	16.80	16.69	13.70	5.46	15.60	3.34	17.22	7.48
Visit 3	13.30	4.50	15.80	6.81	15.70	7.82	17.30	6.83

* Indicates statistical significance, *p* < 0.05.

**Table 4:** Urinalysis and thyroid outcomes of subjects across groups.

Outcome Measurement	Group 1 1 Stick		Group 2 2 Sticks		Group 3 3 Sticks		Group 4 Placebo	
	Mean	SD	Mean	SD	Mean	SD	Mean	SD
pH								
Visit 1	6.30	0.82	6.20	0.48	5.95	0.55	6.05	0.64
Visit 2	6.45	0.69	6.70	0.75	6.65	0.63	6.35	0.75
Visit 3	6.60	0.99	6.40	0.70	6.40	0.84	6.15	0.63
Urobilinogen (mg/dL)								
Semi Qn								
Visit 1	0.28	0.25	0.28	0.25	0.36	0.34	0.20	0.00
Visit 2	0.28	0.25	0.20	0.00	0.28	0.25	0.36	0.34
Visit 3	0.20	0.00	0.20	0.00	0.28	0.25	0.20	0.00
TSH (uIU/mL)								
Visit 1	1.72	0.46	2.18	0.93	1.75	0.87	2.04	0.96
Visit 2	1.66	0.35	2.14	0.94	1.77	0.82	1.64	0.66
Visit 3	1.72	1.04	2.34	0.87	1.57	0.53	2.25	1.06
Thyroxine (μg/dL)								
Visit 1	7.20	0.74	6.75	1.024	7.90	1.43	6.65 *	1.44
Visit 2	7.50	0.66	7.08	1.08	8.38	1.93	6.38 *	1.14
Visit 3	7.29	0.89	7.19	0.99	8.25	1.72	6.65 *	1.23
Triiodothyronine (ng/dL)								
Visit 1	126.60	19.35	115.90	25.17	118.90	21.26	105.60	18.43
Visit 2	121.60	19.81	113.90	26.04	123.10	24.68	104.89	29.81
Visit 3	126.50	16.44	115.40	29.93	129.70	32.99	108.60	20.86

* Indicates statistical significance, *p* < 0.05. Thyroxine, difference between Groups 3 and 4 (*p* = 0.033).

**Table 5:** Average tolerability scores of subjects across groups.

Outcome Measurement	Group 1 1 Stick		Group 2 2 Sticks		Group 3 3 Sticks		Group 4 Placebo	
	Mean	SD	Mean	SD	Mean	SD	Mean	SD
Bloating								
Visit 1	2.72	2.61	0.65	0.83	1.31	1.00	0.96	0.68
Visit 2	1.25	1.68	1.71	2.13	1.82	1.93	0.77	0.73
Visit 3	1.46	1.76	2.49	3.05	1.90	1.28	0.53	0.58
Constipation								
Visit 1	0.48	0.69	0.11	0.23	0.62	1.12	0.48	0.40
Visit 2	0.17	0.27	0.18	0.41	0.38	0.80	0.44	0.33
Visit 3	0.31	0.47	0.77	1.63	0.61	1.21	0.22	0.21
Diarrhea								
Visit 1	0.00	0.00	0.26	0.66	0.01	0.04	0.43	0.68
Visit 2	0.14	0.42	0.27	0.49	0.25	0.49	1.13	2.50
Visit 3	0.46	0.71	0.49	0.85	0.31	0.56	0.82	1.48
Frequent Urination								
Visit 1	1.66	2.08	0.77	0.89	2.08	2.64	1.41	1.63
Visit 2	2.16	2.03	2.13	2.13	3.79	2.24	1.93	1.72
Visit 3	1.64	1.24	2.23	2.60	2.99	2.65	1.54	1.50
Gas								
Visit 1	0.91	1.23	1.26	1.52	1.36	1.05	0.95	0.85
Visit 2	1.16	1.23	1.91	1.85	1.77	1.46	1.12	1.12
Visit 3	1.00	1.44	1.96	2.51	1.71	1.36	0.73	0.75
Nausea								
Visit 1	0.48	1.09	0.03	0.08	0.32	0.73	0.26	0.40
Visit 2	0.41	0.60	0.19	0.43	0.24	0.41	0.65	1.28
Visit 3	0.01	0.04	0.56	0.72	0.39	0.53	0.22	0.37
Stomach Fullness								
Visit 1	2.37	2.04	2.52	2.43	2.38	2.30	1.88	2.06
Visit 2	1.77	2.19	2.53	2.46	3.50	2.46	0.58	0.65
Visit 3	1.65	2.22	3.08	2.60	2.86	2.12	0.67	0.85
Abdominal Cramping								
Visit 1	0.29	0.77	0.11	0.15	0.32	0.52	0.16	0.24
Visit 2	0.08	0.21	0.22	0.52	0.98	1.23	0.16	0.24
Visit 3	0.12	0.37	1.42	2.19	0.47	0.84	0.26	0.42
Total Tolerability Score								
Visit 1	8.46	7.23	5.71	5.12	8.40	5.21	6.53	4.20
Visit 2	7.13	5.05	9.12	8.45	12.73	7.63	6.78	4.77
Visit 3	6.65	5.60	13.26	14.04	11.23	5.72	4.99	3.94

Total tolerability score, bloating, abdominal cramping; interactions = significant interactions between treatment and time (*p* = 0.044, 0.039, and 0.010, respectively); frequent urination = visit 1 and 2 differ with time.

**Table 6:** Average daily nutritional outcomes for subjects.

Outcome Measurement	Group 1 1 Stick		Group 2 2 Sticks		Group 3 3 Sticks		Group 4 Placebo	
	Mean	SD	Mean	SD	Mean	SD	Mean	SD
Calories (kcal)								
Visit 1	1811.32	483.12	2058.89	569.26	2176.41	408.84	1838.66	681.52
Visit 2	1549.55	771.32	1837.10	401.43	2010.53	473.57	1798.52	926.98
Visit 3	1643.73	618.03	1833.16	511.07	2101.54	458.28	1752.25	702.00
Protein (g)								
Visit 1	85.69	34.23	94.80	25.10	90.63	23.67	87.73	51.72
Visit 2	83.57	45.73	82.43	20.20	82.58	26.71	84.60	53.89
Visit 3	80.29	41.46	86.53	31.77	93.88	32.04	80.71	47.62
Carbohydrates (g)								
Visit 1	209.25	50.68	212.86	46.77	250.54	60.74	202.712	97.53
Visit 2	168.88	81.73	207.41	59.29	248.12	61.95	196.92	113.05
Visit 3	168.87	74.06	202.96	58.95	237.70	75.15	207.25	78.30
Total Fiber (g)								
Visit 1	15.48	5.91	17.34	7.06	20.36	6.39	17.02	8.47
Visit 2	11.66	6.25	16.45	7.46	20.48	9.40	17.36	11.17
Visit 3	13.48	7.58	17.27	7.34	19.05	8.78	16.22	5.95
Sugar (g)								
Visit 1	61.51	24.17	57.87	24.91	89.75	38.70	63.80	30.86
Visit 2	49.90	39.50	62.21	40.38	85.00	36.32	61.22	46.48
Visit 3	53.14	35.30	58.00	31.21	77.16	34.66	66.75	39.48
Fat (g)								
Visit 1	73.07 *	23.55	91.96 *	38.00	93.18 *	22.73	78.20 *	30.95
Visit 2	58.80	32.76	77.09	18.00	77.17	23.35	83.01	56.52
Visit 3	73.37	29.34	75.52	22.75	87.81	26.32	68.60	36.42
Cholesterol (mg)								
Visit 1	235.98 *	120.80	420.01 *	180.52	356.29 *	163.93	239.46 *	155.46
Visit 2	264.22	167.35	309.58	202.20	234.48	127.47	290.97	196.64
Visit 3	296.89	174.16	341.68	164.09	312.75	150.70	193.40	116.12
Vitamin D (IU)								
Visit 1	121.92	190.38	134.86	106.08	91.85	73.19	65.31	46.75
Visit 2	55.34	52.54	72.68	51.60	111.93	82.64	40.69	41.26
Visit 3	113.76	199.39	61.33	43.01	126.99	98.82	57.29	44.23
Calcium (mg)								
Visit 1	499.56	260.01	703.65	200.18	740.71	240.57	570.17	131.86
Visit 2	588.06	320.39	538.45	232.41	785.67	281.74	608.74	304.24
Visit 3	534.67	268.35	619.04	252.95	811.28	400.45	550.80	207.29
Sodium (mg)								
Visit 1	3271.86	1375.55	3587.54	1375.55	2766.89	565.04	2966.95	987.27
Visit 2	2923.66	1130.18	3781.51	1151.22	4353.41	905.99	3044.36	1262.90
Visit 3	3186.51	1087.90	3539.12	1174.74	3983.96	1107.54	2823.31	1336.65

* Indicates statistical significance, *p* < 0.05. For fat and cholesterol, visit 1 different from all other time points.

## Data Availability

The data presented in this study may be available on request from the corresponding author due to the privacy agreement with the funder.

## References

[1] Perrier E.T., Armstrong L.E., Bottin J.H., Clark W.F., Dolci A., Guelinckx I., Iroz A., Kavouras S.A., Lang F., Lieberman H.R. (2021). Hydration for Health Hypothesis: A Narrative Review of Supporting Evidence. Eur. J. Nutr..

[2] Perrier E.T. (2017). Shifting Focus: From Hydration for Performance to Hydration for Health. Ann. Nutr. Metab..

[3] Nuccio R.P., Barnes K.A., Carter J.M., Baker L.B. (2017). Fluid Balance in Team Sport Athletes and the Effect of Hypohydration on Cognitive, Technical, and Physical Performance. Sports Med..

[4] Kenefick R.W. (2018). Drinking Strategies: Planned Drinking Versus Drinking to Thirst. Sports Med..

[5] Hellier M.D., Thirumalai C., Holdsworth C.D. (1973). The Effect of Amino Acids and Dipeptides on Sodium and Water Absorption in Man. Gut.

[6] Desjeux J.-F. (1977). Effects of Sugars and Amino Acids on Sodium Movement Across Small Intestine. Arch. Pediatr. Adolesc. Med..

[7] Wu G. (2009). Amino acids: Metabolism, functions, and nutrition. Amino Acids.

[8] Tai C.-Y., Joy J.M., Falcone P.H., Carson L.R., Mosman M.M., Straight J.L., Oury S.L., Mendez C., Loveridge N.J., Kim M.P. (2014). An Amino Acid-Electrolyte Beverage May Increase Cellular Rehydration Relative to Carbohydrate-Electrolyte and Flavored Water Beverages. Nutr. J..

[9] Clarke M.M., Stanhewicz A.E., Wolf S.T., Cheuvront S.N., Kenefick R.W., Kenney W.L. (2019). A Randomized Trial to Assess Beverage Hydration Index in Healthy Older Adults. Am. J. Clin. Nutr..

[10] Maughan R.J., Watson P., Cordery P.A., Walsh N.P., Oliver S.J., Dolci A., Rodriguez-Sanchez N., Galloway S.D. (2016). A Randomized Trial to Assess the Potential of Different Beverages to Affect Hydration Status: Development of a Beverage Hydration Index. Am. J. Clin. Nutr..

[11] Williams M. (2005). Dietary Supplements and Sports Performance: Amino Acids. J. Int. Soc. Sports Nutr..

[12] Gervasi M., Sisti D., Amatori S., Donati Zeppa S., Annibalini G., Piccoli G., Vallorani L., Benelli P., Rocchi M.B.L., Barbieri E. (2020). Effects of a Commercially Available Branched-Chain Amino Acid-Alanine-Carbohydrate-Based Sports Supplement on Perceived Exertion and Performance in High Intensity Endurance Cycling Tests. J. Int. Soc. Sports Nutr..

[13] Kreider R.B., Miriel V., Bertun E. (1993). Amino Acid Supplementation and Exercise Performance: Analysis of the Proposed Ergogenic Value. Sports Med..

[14] Carretero-Gómez J., Arévalo Lorido J.C., Gómez Huelgas R., De Escalante Yangüela B., Gracia Tello B., Pérez Belmonte L., Ena Muñoz J. (2018). SEMI Working Group of Diabetes and Obesity Hydration and Obesity among Outpatient-Based Population: H2Ob Study. J. Investig. Med..

[15] Laja García A.I., Moráis-Moreno C., Samaniego-Vaesken M.d.L., Puga A.M., Varela-Moreiras G., Partearroyo T. (2019). Association between Hydration Status and Body Composition in Healthy Adolescents from Spain. Nutrients.

[16] Epstein Y., Armstrong L.E. (1999). Fluid-Electrolyte Balance during Labor and Exercise: Concepts and Misconceptions. Int. J. Sport. Nutr..

[17] Nerbass F.B., Pecoits-Filho R., Clark W.F., Sontrop J.M., McIntyre C.W., Moist L. (2017). Occupational Heat Stress and Kidney Health: From Farms to Factories. Kidney Int. Rep..

[18] Sollanek K.J., Tsurumoto M., Vidyasagar S., Kenefick R.W., Cheuvront S.N. (2018). Neither Body Mass nor Sex Influences Beverage Hydration Index Outcomes during Randomized Trial When Comparing 3 Commercial Beverages. Am. J. Clin. Nutr..

[19] Wolf S.T., Stanhewicz A.E., Clarke M.M., Cheuvront S.N., Kenefick R.W., Kenney W.L. (2019). Age-Related Differences in Water and Sodium Handling after Commercial Hydration Beverage Ingestion. J. Appl. Physiol..

[20] Park S.Y., Park S.E., Jung S.W., Jin H.S., Park I.B., Ahn S.V., Lee S. (2017). Free Triiodothyronine/Free Thyroxine Ratio Rather than Thyrotropin Is More Associated with Metabolic Parameters in Healthy Euthyroid Adult Subjects. Clin. Endocrinol..

[21] Gomes-Lima C., Wartofsky L., Burman K. (2019). Can Reverse T3 Assay Be Employed to Guide T4 vs. T4/T3 Therapy in Hypothyroidism?. Front. Endocrinol..

[22] Kellum J.A. (2000). Determinants of Blood pH in Health and Disease. Crit. Care.

[23] Han Y., Choi B., Kim S., Kim S.-B., Kim Y., Kwon E.-Y., Choi M.-S. (2018). Gastrointestinal Tolerance of D-Allulose in Healthy and Young Adults. A Non-Randomized Controlled Trial. Nutrients.

